# Childhood Unintentional Injuries: Need for a Community-Based Home Injury Risk Assessments in Pakistan

**DOI:** 10.1155/2012/203204

**Published:** 2012-04-10

**Authors:** Adnan A. Hyder, Aruna Chandran, Uzma Rahim Khan, Nukhba Zia, Cheng-Ming Huang, Sarah Stewart de Ramirez, Junaid Razzak

**Affiliations:** ^1^International Injury Research Unit (IIRU), Department of International Health, Johns Hopkins University Bloomberg School of Public Health, Baltimore, MD 21205, USA; ^2^Department of Emergency Medicine, Aga Khan University, Karachi 74800, Pakistan

## Abstract

*Background*. A substantial proportion of the annual 875,000 childhood unintentional injury deaths occur in the home. Very few printed tools are available in South Asia for disseminating home injury prevention information. *Methods*. Three tools were planned: an injury hazard assessment tool appropriate for a developing country setting, an educational pamphlet highlighting strategies for reducing home injury hazards, and an in-home safety tutorial program to be delivered by a trained community health worker. *Results*. The three tools were successfully developed. Two intervention neighborhoods in Karachi, Pakistan, were mapped. The tools were pretested in this local setting and are now ready for pilot testing in an intervention study. *Conclusion*. Planning for an innovative, community-based pilot study takes considerable time and effort in a low-income setting like Pakistan. The primary outcome of the pre-testing phase of the study was the development of three important tools geared for low-income housing communities in Pakistan.

## 1. Background

Unintentional injuries are major causes of mortality and morbidity in children, resulting in over 875,000 deaths annually in children <18 years of age [1–3]. Among children <5 years of age, injuries are the leading cause of death after the first birthday. Additionally, millions of children require medical care with hospital admission for nonfatal injuries and are often left with lifelong disabilities [1]. Although unintentional injury is a major contributor to mortality worldwide, the burden is unequally distributed between low- and middle-income countries (LMICs) and high-income countries (HICs). The mortality rate from unintentional injuries in LMIC is nearly double that in HIC (65 versus 35 per 100,000), while the rate of disability-adjusted life-years (DALYs) is three times as high in LMIC as compared with HIC (2,398 versus 774 per 100,000) [4]. The disproportionate burden of unintentional injuries borne by LMIC is due in large part to high risks, inadequate preventive measures, and a lack of access to appropriate and timely medical care [5].

A substantial proportion of childhood unintentional injuries occur in the home, as a result of the relatively long period of time young children spend in the home and the many potential sources of hazards that are present [6]. The nature of childhood injuries occurring in the household has been well described in HICs but they are less well understood in LMICs [7, 8]. However, a recent study in Nigeria found that 92.5% of childhood burns occurred in a domestic setting, suggesting that the prevention of childhood injuries in the home environment is a key area for future research [9]. Challenging living conditions such as poor housing infrastructure, lack of barriers to cooking or washing areas, inadequate recreational space, use of open fires and paraffin stoves, and a lack of safe storage for harmful substances are among the hazards that place young children in LMICs at risk for burns, poisoning, and falls [10–12].

Despite these substantial risks, the majority of household injuries can be prevented. In developed countries, a variety of preventive approaches have been shown to be effective including legislative measures, modification of the home environment with provision of safety equipment, and focused injury prevention counseling [13–17]. In addition, studies utilizing home visitation programs that include education and advice have been undertaken to gauge the impact of such interventions on injury reduction [18, 19]. Results suggested that home visits were flexible and acceptable to families as well as helpful in increasing household members' knowledge, attitude, and behavior regarding home safety. Additionally, a meta-analysis of home safety education and safety equipment revealed that home safety education, particularly when coupled with the provision of safety equipment, was effective in increasing a range of safety practices associated with the prevention of burns, poisoning, and fall-related injuries [16]. These studies offer strong evidence that home visitations can reduce childhood injuries in HICs; however, additional research is needed to assess the efficacy and acceptability of childhood home safety programs in LMICs.

Preliminary evidence suggests that a home-safety education program may be possible in LMIC. A study of pediatric scalding prevention in South Africa identified categories of prevention measures that included enhancements to the safety of the home environment, changes to practice, and improvements to individual competence [20]. In addition, a recent study of perceptions about childhood burns in rural Bangladesh suggests that a safety education program could offer an effective means of increasing knowledge and practice [21]. However, further research is needed to establish a means for sharing known, proven hazard reduction strategies tailored to these communities.

While children are universally vulnerable to injuries, the social, political, and economic environment shapes the nature and extent of injury risk [1]. Thus, effective solutions require that intervention materials be tailored to the local context. While a number of household injury assessment tools have been utilized in HICs, differences in the nature of household risks make the direct transfer of such tools to LMIC settings inappropriate. A well-designed tool is essential for the systematic comparison of household injury risk over time and between households [22–24]. Moreover, the lack of research regarding the most effective method for dissemination of home injury risk and potential prevention information in LMIC has resulted in limited ways for health professionals to share knowledge with parents [1]. There are no predesigned pamphlets or information sheets available in this setting, in contrast to the abundance of such materials available to practitioners in HICs.

This paper introduces a study that aims to address this research gap. The study focuses on the development of two different hazard reduction information tools—an educational pamphlet and an in-home tutorial—to explore their use in a community-based LMIC setting. It also develops a hazard mapping tool for low-income household settings. This paper is based on the preliminary phase of the study and describes the approach and development of these tools in preparation for a pilot study to test their implementation and acceptability in Pakistan.

## 2. Methods

### 2.1. Conceptual Framework

Childhood injury risk has been assessed within a number of conceptual frameworks; however, Morrongiello et al. have suggested that the “process analytic approach” originally proposed by Peterson et al. offers the greatest insight into the determinants of injury [25, 26]. The “process analytic approach” is action oriented and allows for an analysis of multiple risk factors; as a result, it enables implementers to focus interventions on those factors most closely associated with childhood injury [25]. Injury risk has been shown to be an interaction between the home environment and a child's temperament/behavior; use of the process in a study of home injury risk among toddlers demonstrated that modifying the home environment significantly reduced the risk of home injury among children [27].

**Figure 1:**
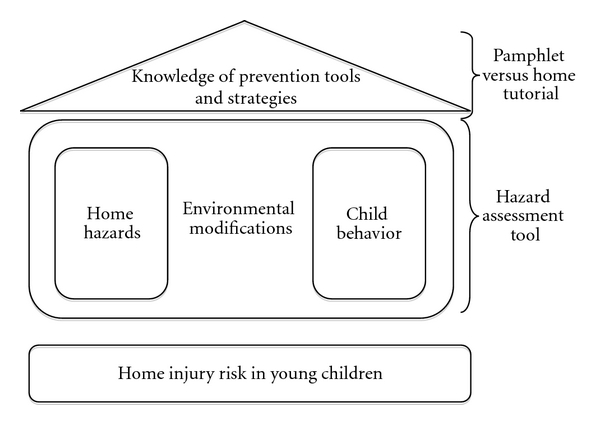
Schematic framework for home injury hazard assessment and reduction.

We build on this existing framework to define a conceptual framework for our study (Figure 1). Building on the base of home injury risk in young children, we propose to show that the home environment and the child's behavior interact through environmental modifications to further define injury risk. Our hazard assessment tool attempts to quantify this risk. Overlaying this is the caregiver's knowledge of prevention tools/strategies and they might modify the environment. We hope to test the best way to strengthen the role of the caregiver in the future by comparing two different ways of educating them (an educational pamphlet and a home-based tutorial) in an LMIC setting.

### 2.2. Proposed Pilot Study in Pakistan

Pakistan is a low-income country with a per capita Gross National Income of just over US $2,000. A study based on the National Health Survey of Pakistan reported an overall annual incidence of unintentional injuries in children <5 years of age of 47.8 per 100,000 (95% CI: 36.6, 59.0), resulting in approximately 1.1 million unintentional injuries per year [28]. Additionally, a recent study of unintentional injuries in children aged 1 to 8 years in two suburban and rural communities in Pakistan found that the majority of injuries occurred inside the home [29]. Studies are needed in Pakistan to address the gap in knowledge regarding the burden and strategies for reduction of childhood (ages 12 through 59 months) injury risks in the home. We introduce such a study—Global Childhood Unintentional Injury Surveillance, Pakistan (GCUIS-Pak)—which is a continuation of the “global” work done previously in multiple countries [6]. We focus this section on the initial pretesting phase of GCUIS-Pak work in Karachi, Pakistan's largest city with an estimated population of 15 million.

The aims of this phase of GCUIS-Pak were threefold: first, to develop and pretest an injury hazard assessment tool appropriate for a low-income, urban, developing country setting, second, to develop and pretest an educational pamphlet addressing the importance of injury hazards in the home and promoting methods to reduce them, and finally, to develop and pretest a home-based tutorial program for its feasibility and acceptability as a means of disseminating home safety information in Pakistan.

For the purposes of this study, two neighborhoods were identified within a low-income government housing community in Karachi. This community was selected on the basis of a lower middle-income status of residents, homes with permanent structures, relatively high literacy level of residents, and ease of access from the local research institution. The majority of residents in the community are young families who reside in the neighborhood for the duration of the primary provider's government service job. The housing units are comprised of 2 to 4 small rooms with an outdoor enclosed courtyard. The majority of houses have a separate kitchen and bathroom, which are located off the courtyard, and some families have chickens, parrots, or other small animals that are kept in cages in the courtyard. There are schools, stores, and medical care facilities located within neighborhoods. While very few residents have cars, the majority own a small scooter or motorcycle as their form of transportation.

The two neighborhoods selected for the study were mapped, and families with at least one child between the ages of 12 and 59 months were identified for recruitment. The neighborhoods are located nearly 1/4 mile apart and separated by two large busy streets. Families in the first neighborhood will receive an in-home tutorial, while those in the second neighborhood will receive an educational pamphlet. A follow-up assessment of each household will be conducted approximately 3 to 4 months after the initial visit. In the interim, a qualitative study will be undertaken in a smaller sample of each neighborhood to better understand the feasibility and acceptability of each tool in this community. Further details of the methods will be provided in the paper presenting the quantitative results when the study is completed. The study has been approved by the ethics committees (Institutional Review Boards) at Aga Khan University, Pakistan, and Johns Hopkins Bloomberg School of Public Health, USA.

## 3. Results

**Figure 2:**
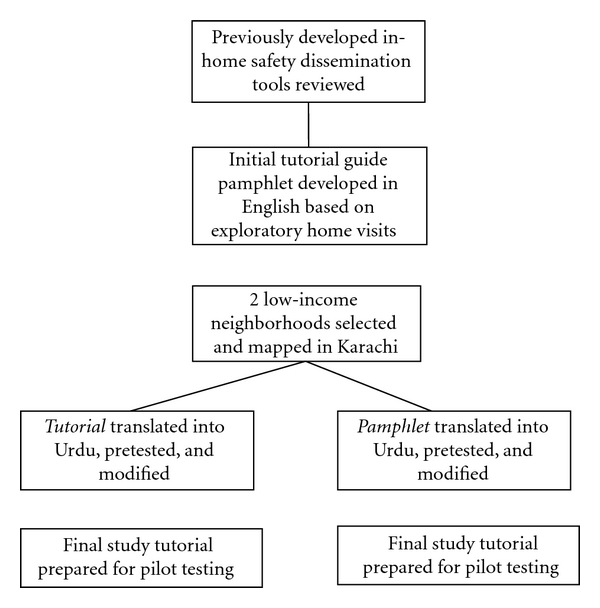
Process of development of new tools for child injury risk assessment.

In preparation for an eventual pilot intervention study, the following tools were developed: (1) a home hazard risk assessment tool, (2) an educational pamphlet, and (3) an in-home tutorial guide. The flow of tool development is shown in Figure 2. Each was developed with local involvement, pretested, modified, and then finalized for use in the study (Table 1).

### 3.1. Home Hazard Assessment Tool

Home hazard assessment tools are needed to identify and quantify existing child injury risks. Such tools also serve as an effective means to monitor potential changes in risk resulting from interventions. While a number of assessment tools exist from HICs, they have not been formulated for LMICs [22–24]. As a result, an assessment tool was developed based on a review of existing tools and expert consultation with local pediatric/emergency department providers and local injury prevention experts/researchers. The Home Hazard Assessment Tool (HHAT) covers four domains including demographic information, recent injury history, description of the house, and a checklist for the identification of hazards by area or room of the home (Table 2). The checklist areas included the kitchen, bath area, living/sleeping area, courtyard/rooftop, and the outdoors immediately surrounding the home. 

The goal of the tool was to enable a community health worker (CHW) to inspect a household and document risks for injuries to young children (12 to 59 months of age) within the home. The HHAT tool was translated into the local language (Urdu) and pretested in 15 households of a community (separate from the chosen study sites) to test for ease of use, relevance, and understanding (Table 1). It was revised and finalized for use in the pilot study. Following informed consent, CHWs will use the newly developed HHAT tool to inspect each room of a house and document risk by observation, rather than on the basis of reporting by a household member. In these initial safety assessments, the caretaker of the child will serve as the respondent for the assessment, and the collected data will provide the baseline assessment.

### 3.2. Educational Pamphlet

The goal of the educational pamphlet was to provide childhood safety prevention information in a format not requiring the presence of a health practitioner for understanding or use. The study team reviewed existing injury prevention materials from HICs, including pamphlets and fact sheets on burns, falls, and drowning, similar to those available from the United States' Centers for Disease Control and Prevention [30]. Targeting key types of childhood injuries, teaching points were identified and categorized by household area (living room, kitchen, etc.) with the goal of promoting behavior change by first providing information (*predisposing* factors), and then making suggestions in a useable format (*enabling* factors) in line with our conceptual framework (Figure 1) [31]. This approach is also consistent with previous work in child injury prevention that focuses on enabling families to make changes to risk factors [16, 18, 19].

**Figure 3:**
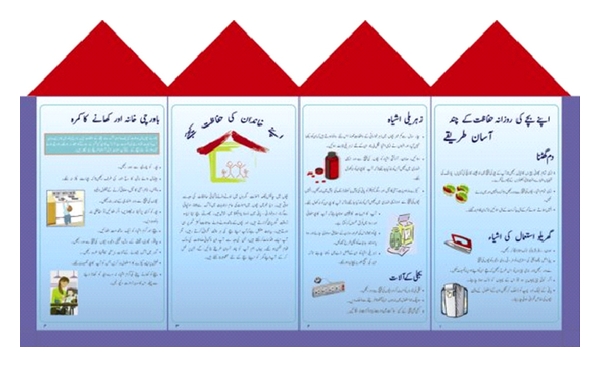
Example selections from Educational Pamphlet in Urdu.

Exploratory home visits were first conducted to understand the basic layout of the homes in these types of study communities. In addition, study staff gained a better understanding of the literacy level of home occupants in the chosen study areas. After reviewing and compiling the major safety tips given in existing pamphlets and written resources, a composite pamphlet was drafted in English and then translated into Urdu and tailored to the local setting with commonly used vernacular and phrasing. This draft pamphlet was discussed with families in 10 home visits to ensure that messages were relevant to the local environment. A revised version was then drafted, and a local graphic designer was hired to depict safety scenes applicable to the local surroundings. Finally, the Urdu pamphlet was pretested in 16 households in one community similar to the chosen study sites. Suggestions regarding wording, pictures, and layout were incorporated into the final pamphlet to be used in the study (Figure 3). 

### 3.3. In-Home Tutorial Guide

The goal of the home safety tutorial guide was to test an interactive tool that allows a CHW to provide home injury risk information and prevention ideas while walking through the house with the participant. This approach was based on similar tutorials available for HICs, often dubbed “Safety Checklists”, which are organized by room or area of the home. A number of these checklists and tutorials were reviewed including home safety checklists from *KidsHealth.org* and the guides at the *U.S. Home Safety Council* [32, 33]. In each room in the home, the CHW and the household member would need to work together to identify specific examples of safety or risks. The CHW could then discuss with the participants inexpensive and potentially simple ways in which identified risks could be altered to make the home potentially safer for their child.

**Figure 4:**
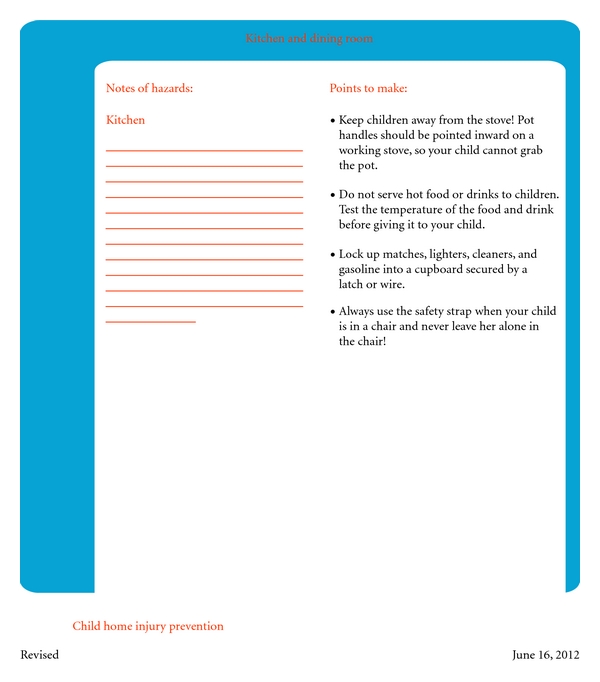
Example from In-Home Tutorial Guide in English.

An initial tutorial was developed in English based on a review of the literature and expert input from local pediatric/emergency department providers and local injury prevention experts/researchers. Because such an approach was new to the communities in Pakistan, the English version of the tutorial was used to gather feedback from 39 home visits by local investigators who speak both English and Urdu (Figure 4). Valuable suggestions were obtained on tutorial content and style of delivery, which were then used for revision and translation into Urdu. The Urdu tutorial was pretested in 15 homes in a community similar to the study communities (Table 1). Suggestions regarding wording, discussion points, and layout were used to develop the final version, which is now ready for the pilot study.

## 4. Discussion

It is unfortunate that despite the burden of unintentional injuries in young children in LMICs, and the known significance of home injury risks, there are no existing well-adapted tools to disseminate the importance of home injury risks or how these hazards might be modified. Information can be disseminated through passive written materials or active in-home tutorials, both of which are utilized in HICs. Yet few such materials have been tailored to LMIC settings; therefore, this pilot (GCUIS-Pak) study aims to develop and test such materials in a study. This approach is consistent with well-established practices in other areas of child health. For example, CHWs have been used to increase coverage of infectious disease interventions for decades in low-income settings [34, 35]. Furthermore, delivery of educational messages and self-help instructions has been widely used in child survival programs [36]. The current study offers a novel approach by adapting these principles for child injury prevention in the developing world.

Planning for an innovative, community-based pilot study takes considerable time and effort in a low-income setting like Pakistan. This paper has introduced the proposed pilot study and provides an initial report of the first phase. The primary outcome of the pretesting phase of the study was the development of three important tools geared for low-income housing communities in Pakistan: (1) an assessment tool to quantify the in-home childhood injury hazards, (2) an educational pamphlet outlining important injury hazard and prevention information geared towards children 12 to 59 months of age, and (3) an in-home tutorial guide focused on providing information on low-cost injury prevention techniques for children ages 12 to 59 months. Of note, community-based participatory qualitative research methodology was not used in the development of these tools. Instead, a process of adapting existing widely utilized tools from high-income countries to the local setting was employed in order to create these pilot materials. This sets up a unique opportunity to engage in a well-constructed qualitative study with the community after they have had exposure to the three tools in a well-structured study setting.

These materials are among the first of their kind tailored to a South Asian, LMIC setting. Further study is needed to understand their effectiveness, as well as the feasibility and acceptability of their use. The pilot study is currently underway in these two low-income neighborhoods in Karachi to respond to these questions. This paper describes these tools to also provide an opportunity for modification of these tools to other LMIC settings and the chance to expand upon existing knowledge regarding home injury risk reduction in low-income settings.

This study serves as an excellent opportunity for capacity building on multiple levels. Junior investigators in Pakistan have the opportunity to take the lead on various aspects of this project under the guidance and mentorship of senior investigators in both Pakistan (at Aga Khan University) and USA (Johns Hopkins International Injury Research Unit). Pakistani investigators managed all aspects of this project, from creation of the study tools, pretesting, and finalization of materials; additionally, these same investigators will be integrally involved in the intervention study at all phases. The collaboration between a US institution and a Pakistani university helped build the foundation for future child injuries research. Moreover, the pilot study, though limited in scale, has already helped raise the profile of child injury research at both institutions. Lastly, this project builds on the existing relationships between an established international injury research center in the USA (http://www.jhsph.edu/iiru/) and a strong Department of Emergency Medicine in Pakistan (http://hospitals.aku.edu/karachi/hospitaldepartments/emergencymedicine/Pages/departmentofemergencymedicine.aspx), demonstrating an interdisciplinary approach to child injury research.

The pilot intervention study has been initiated in Pakistan and, despite the security challenges, continues at a good pace in 2011. The results of the main study will be reported by early 2012 and hope to influence larger and more robust studies to evaluate the impact of CHW-based approaches to child injury prevention. Such studies are vital to provide effective and sustainable interventions to address the burden of childhood injuries in Pakistan and the developing world.

## Figures and Tables

**Table 1:** Pretesting phase for child injury risk tools in Pakistan.

Tasks	HHAT	Pamphlet	Tutorial
Initial draft prepared based on	(i) Review of the published literature on child injuries, and home risk reductions (ii) Review of existing print materials from international agencies (World Health Organization, Centers for Disease Control, Poison Control Centers, etc.) (iii) Expert opinion from healthcare providers in USA and Pakistan (iv) Selected community health workers in Pakistan		
Formulate Test (number of households)	AKU	10	39
Pretest (number of households)	15	16	15
Time to completion (months)	3 months	3 months	3 months

*HHAT: Home Hazard Assessment Tool; AKU: Aga Khan University, Pakistan.

**Table 2:** Example from home hazard assessment tool: kitchen area.

Kitchen	Yes	No
(1) Is the stove within reach of the child?	□	□
(2) Are matches/lighter/cookingfluids (i.e., paraffin or kerosene) within reach of the child?	□	□
(3) Are cleaning supplies/chemicals within reach of the child?	□	□
(4) Are there any knives or sharp objects within reach of the child?	□	□
(5) Is there any open fire/fireplace within reach of the child?	□	□
(6) Is there a fire extinguisher or bag of sand kept in the kitchen?	□	□
(7) Are cupboards with cooking fluids, cleaning supplies, knives, and matches secured or locked?	□	□
(8) Are lighter/cooking fluids kept in nonoriginal or non labeled containers?	□	□
(9) Type of drinking water glasses at home (circle) (a) Steel (b) Plastic (c) Glass (d) Other		
